# Real-time Colorimetric Quantitative Molecular Detection of Infectious Diseases on Smartphone-based Diagnostic Platform

**DOI:** 10.1038/s41598-020-65899-w

**Published:** 2020-06-02

**Authors:** Kun Yin, Vikram Pandian, Karteek Kadimisetty, Xin Zhang, Carlos Ruiz, Kumarasen Cooper, Changchun Liu

**Affiliations:** 1 Department of Biomedical Engineering, University of Connecticut Health Center, 263 Farmington Avenue, Farmington, CT 06030 USA; 2 Department of Mechanical Engineering and Applied Mechanics, University of Pennsylvania, 220 South 33rd St., Philadelphia, Pennsylvania 19104-6315 USA; 3 Department of Pathology and Laboratory Medicine, University of Pennsylvania, 3400 Spruce St., Philadelphia, PA 19104 USA

**Keywords:** Diagnosis, Analytical chemistry, Biomedical engineering

## Abstract

Rapid diagnostics of infectious diseases and accurate identification of their causative pathogens play a crucial role in disease prevention, monitoring, and treatment. Conventional molecular detection of infectious pathogens requires expensive equipment and well-trained personnel, thus limiting its use in centralized clinical laboratories. To address this challenge, a portable smartphone-based quantitative molecular detection platform, termed “smart connected pathogen tracer” (SCPT), has been developed for pathogen monitoring and disease surveillance. The platform takes advantage of synergistically enhanced colorimetric loop-mediated isothermal amplification (LAMP) assay and smartphone-based color analysis, enabling simple, rapid and reliable nucleic acid quantification without need for expensive fluorescence detection equipment. The SCPT platform has been successfully applied to quantitatively detect: i) HPV DNA in saliva and clinical vaginal swab samples, and ii) HIV RNA in plasma samples with comparable sensitivity to state-of-art machine. It has also been demonstrated for disease spatiotemporal mapping and pathogen tracking by wireless connection and web-based surveillance. Such simple, cost-affordable, portable molecular detection platform has great potential for on-site early disease detection, remote healthcare monitoring, and epidemic surveillance.

## Introduction

Infectious diseases have become the leading causes of death and posed a considerable threat to global health^1,2^. Lack of access to simple, rapid and low-cost diagnostic technologies for infectious disease detection contributes to enormous burden of infectious diseases globally, especially in resource-limited settings. Nucleic acid-based molecular detection has been widely used for clinical diagnostics, biodefense and molecular biology research due to its high sensitivity, specificity, and flexibility^3–5^. In many disease diagnosis and treatment, quantitative detection of pathogenic nucleic acids is critical to predict disease progression, monitor emergence of drug-resistance, and assess the effectiveness of drug therapy^6–8^. Real-time fluorescence quantitative polymerase chain reaction (qPCR) method is the most commonly used technology for nucleic acid quantification and has been considered as the “gold standard” for many disease diagnostics^9–11^. However, fluorescence qPCR detection typically requires expensive PCR equipment and well-trained personnel, all of which restrict its use in centralized clinical laboratories. Therefore, there is an unmet need for simple, affordable, mobile, quantitative, molecular detection technology that can be easily performed by minimally-trained individuals in resource-limited settings.

Rapid advances in mobile communication and consumer electronics have revolutionized our lives. According to Newzoo, the number of smartphone users across the world will reach to 3.8 billion by 2021^12^. The ubiquitous smartphone with advanced computing capability and built-in functional modules (e.g., smartphone camera, global position system (GPS)) offers unprecedented opportunities in remote diagnostics, disease monitoring, and public health surveillance, creating new paradigms of healthcare, including telemedicine, mobile health (mHealth). In particular, in the context of internet of medical things (IoMT)^13,14^, smartphone-based detection combined with other emerging technologies (e.g., microfluidics, nucleic acid isothermal amplification technology) has present great potential in developing a smart, connected, cost-effective point of care (POC) diagnostic device for use at home, in the field, and at the doctor’s office by ubiquitous internet access.

Recently, several smartphone-based detection platforms have been developed for nucleic acid-based molecular diagnostics^15–20^. For example, smartphone-based fluorescence microplate reader integrated with a fiber-optic bundle has been developed for qualitative detection of nucleic acids by loop mediated isothermal amplification (LAMP)^15^. Fluorescence imaging detection has been adapted for multiplexed testing of different pathogenic nucleic acids by using smartphone along with blue LED light source and fluorescence filter^16^. To eliminate the need for expensive fluorescence optical components (e.g., optical filters, excitation light source), a synergistically enhanced colorimetric LAMP assay has been developed for multiple pathogen detection by using smartphone^20^. However, all of these approaches can only provide qualitative (yes/no) test results. To quantitatively detect nucleic acids, a programmed smartphone was used to monitor bioluminescence in real-time during bioluminescence-based LAMP assay^17^. To generate bioluminescence signals, extra enzymes and substrates (e.g., luciferase, luciferin, ATP sulfurylase) were needed to add into LAMP reaction system, which potentially increases test cost and experimental complexity. In addition, to detect relatively weak bioluminescence signals by smartphone camera, it requires to work in dark room environment (or “black box”) to exclude ambient light interference.

Here, we reported a simple, inexpensive, portable, hue-based quantitative molecular detection platform (termed “smart connected pathogen tracer” (SCPT)) for pathogen detection and connected healthcare monitoring. The platform takes advantage of a colorimetric LAMP assay and smartphone-based real-time color analysis, enabling highly sensitive, cost-effective, quantitative molecular detection at the point of care. Unlike conventional real-time fluorescence detection^15,16^, our platform monitors the color change of colorimetric LAMP assay in real-time, analyzes hue values, and quantifies target nucleic acid directly by an unmodified smartphone. To our best knowledge, it is the first time to directly use an unmodified smartphone to quantitatively detect nucleic acids by real-time colorimetric LAMP assay. As an application demonstration, the platform was successfully used to quantitatively detect HPV in saliva samples/clinical vaginal swab samples, and HIV in plasma sample. Furthermore, quantitative test results can be reported by the smartphone and wirelessly transmitted to a website, together with the testing time and GPS coordinates, allowing real-time pathogen tracking and disease mapping.

## Methods

### Materials and Instruments

KCl, KOH, (NH_4_)_2_SO_4_, Tween 20, Eriochrome black T (EBT), hydroxy naphthol blue (HNB), and Xylidyl Blue 1 (XB 1) were purchased from Sigma-Aldrich. EvaGreen dye was purchased from Biotium. *Bst* 2.0 DNA Polymerase, dNTP, MgSO_4_, and 10X isothermal amplification reaction buffer, were purchased from New England BioLabs (NEB). QIAamp DNeasy Blood and Tissue Kit and Qiagen Viral RNA mini kit were obtained from QIAGEN for HPV 16 DNA and HIV RNA extraction, respectively. PCR primers and LAMP primers were purchased from Integrated DNA Technologies, Inc. Saliva samples were obtained from participants without expectorating or swallowing for 3 to 5 min. Plasma sample was purchased from Innovative Research, Inc. AcroMetrix HIV-1 Control was purchased from Thermo Fisher Scientific. All chemicals used were analytical reagent grade or better. Real-time fluorescence LAMP reaction and real-time fluorescence PCR were carried out on CFX96 Touch Real-Time PCR Detection System (Bio-Rad, USA). Real-time hue-based LAMP assay was performed on the SCPT platform equipped with Galaxy S6 smartphone (Samsung, South Korea). All methods were carried out in accordance with relevant guidelines and regulations.

### LAMP experimental protocol

LAMP primers mix: 40 μL 100 µM FIP/BIP, 20 μL 100 µM LF/LB (or LF), and 5 μL 100 µM F3/B3. 2 mM EBT, HNB, and XB1 dyes were prepared by ddH_2_O. NEB LAMP reaction buffer solution: 1.5 μL 10X isothermal amplification reaction buffer (New England BioLabs Inc), 0.6 μL *Bst* 2.0 DNA polymerase, 2.1 μL dNTP, 0.8 μL LAMP primers mix, 0.9 μL 2 mM EBT, 7.2 μL ddH_2_O and 0.9 μL 100 mM MgSO_4_. Pre-prepared non-buffered LAMP reaction solution: 25 μL 2 M KCl, 1 μL Tween 20, 10 μL 1 M (NH_4_)_2_SO_4_, 140 μL dNTP, 40 μL *Bst* 2.0 DNA Polymerase, 4 μL 1 M KOH, and 280 μL ddH_2_O. Non-buffered LAMP reaction solution^21^: 7.5 μL pre-prepared non-buffered LAMP reaction solution, 0.8 μL LAMP primers mix, 3.6 μL ddH_2_O, 0.9 μL EBT and 1.2 μL 100 mM MgSO_4_. To detect HPV 16, 1 μL sample was added into the non-buffered LAMP reaction solution and incubated at 63 °C. For the detection of HIV RNA using RT-LAMP reaction, 2 U of AMV reverse transcriptase (Invitrogen, Carlsbad, CA) was added in the non-buffered LAMP reaction solution with 1 μL sample and incubated at the same condition. Primers used for HPV16 and HIV isothermal amplification were listed in Table S1.

### Microfluidic chip fabrication and operation

The microfluidic chip body was fabricated by computer numerical control (CNC) machine and assembled by a 250 µm PMMA film top, a 3 mm chip body and a 250 µm PMMA film bottom^22,23^. The microfluidic chip contains four independent LAMP reactors equipped with Qiagen silica membranes. The porous silica membrane is capable of capturing and concentrating nucleic acids from rural samples. For testing, the pathogens in samples (e.g., saliva, plasma, and swab) were first lysed by using Qiagen lysis buffer. Next, the lysate sample was introduced into the microfluidic chip through the inlets. After the sample flew through the Qiagen silica membrane in the reactor, the nucleic acids were captured by the membrane^22,23^. After washing by Qiagen wash buffer 1 (AW1) and Qiagen wash buffer 2 (AW2), respectively, 25 μL of the non-buffered LAMP reaction solution was injected into each reactor. To prevent liquid evaporation, both inlet and outlet of the microfluidic chip were sealed by PCR Sealers tape (Microseal ‘B’ Film) (Bio-Rad). Then, the chip was inserted into the handheld SCPT platform for LAMP amplification.

### Smart connected pathogen tracer platform

The SCPT platform (Figure S1 and Video S1) consists of: i) a portable processor which was powered by battery and provided heat for on-chip isothermal amplification, ii) a disposable microfluidic chip for nucleic acid on-chip extraction and LAMP amplification, and iii) a programmed smartphone for real-time hue-based quantitative molecular detection. The 3D printed processor housed a thin-film heater, a 3.3-V rechargeable lithium-ion battery, an electric circuit board, a plastic optical diffuser, a chip holder and a smartphone adaptor. The custom app was developed with Eclipse Integrated Development Environment (IDE) in Java and Android Developer Tools. It provided an operating instruction to guide the user to run the SCPT platform, allowing the operation by minimally-trained personnel. The app allowed one to set up the number of LAMP reactors and designate their detection area. During real-time colorimetric LAMP detection, the phone camera took an image once every minute for 60 minutes by using built-in camera flash as the illumination source. An optical diffuser was applied to expand the flashlight to uniformly illuminate the microfluidic chip. The images were processed and analyzed in real-time by the smartphone, and the average hue value change of each LAMP reactor was extracted and depicted as a function of time. At the end, the test results obtained by smartphone can be sent to a custom website or cloud server, enabling smart, connected molecular detection.

### Clinical vaginal swab samples preparation

Clinical vaginal swab samples were obtained from the Hospital of the University of Pennsylvania and approved by its ethics committee (IRB protocol #: 829760). The informed consent was obtained from all subjects. The Pap smear tests of the clinical samples were completed at the Hospital of the University of Pennsylvania. The test of clinical swab samples and saliva samples was approved by the University of Connecticut Health Center and the University of Pennsylvania. 200 μL clinical vaginal swab samples were firstly centrifuged at 1000 × g for 5 mins to remove the liquid supernatant. The concentrated cervical cells washed by ddH_2_O for 3 times were resuspended in 200 µL PBS, mixed with 20 µL proteinase K, 200 µL buffer AL (QIAamp DNeasy Blood and Tissue Kit), and then incubated at 56 °C for 10 min. The lysate was mixed with 200 µL ethanol and introduced into the microfluidic chip for nucleic acid extraction and purification^22,23^. The real-time PCR testing of related clinical samples was followed by the previously published method^24^.

## Results

**Figure 1:**
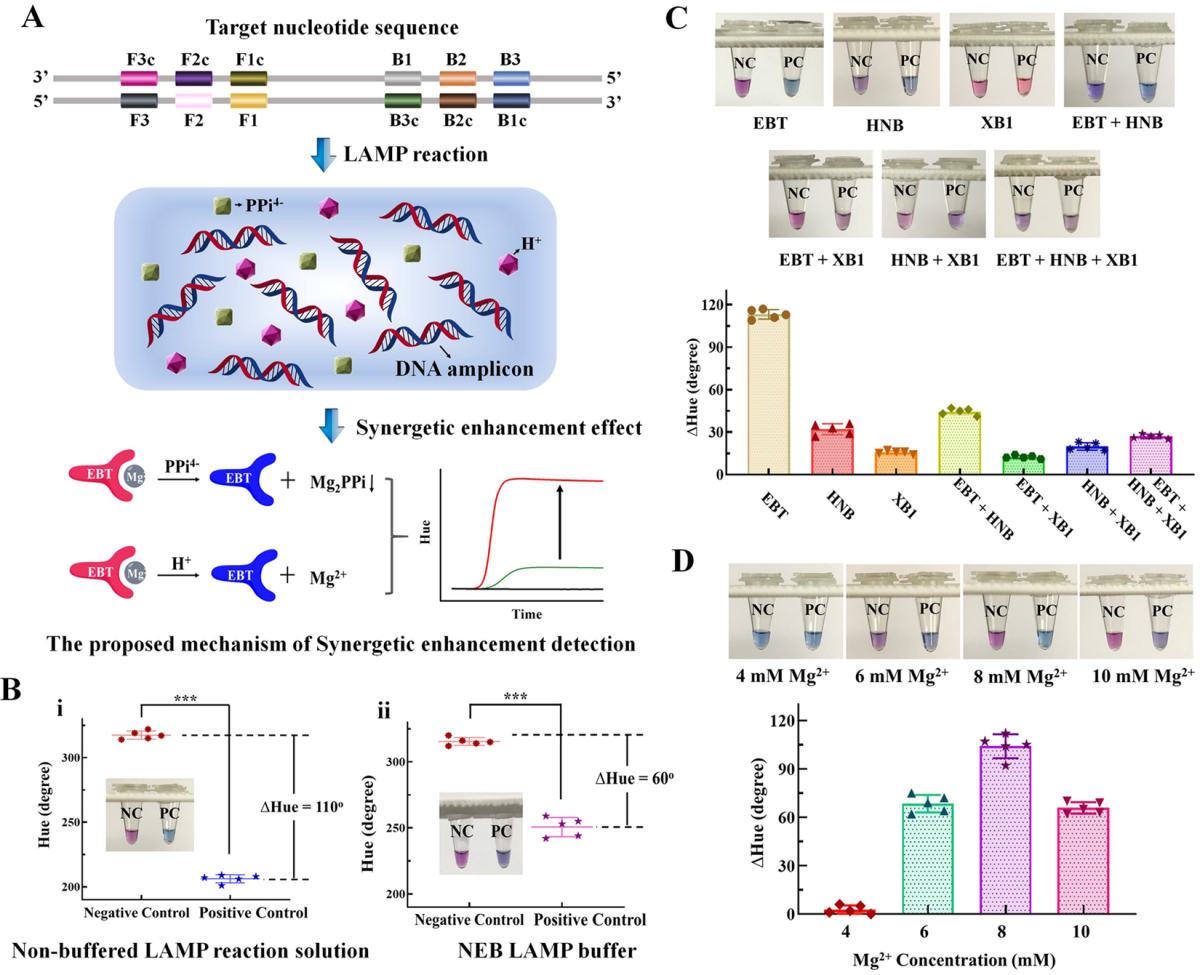
Optimization of real-time colorimetric LAMP assay. (**A**) Detection mechanism of real-time colorimetric LAMP assay based on synergistic enhancement effect of H^+^ and PPi^-^ ions, two byproducts of LAMP reaction. (**B**) Comparison of color change between positive control (PC) (1 × 10^4^ copies HPV 16 DNA template) and negative control (NC) (no DNA template) in colorimetric LAMP assay with: i) non-buffered LAMP reaction solution, and ii) commercially available NEB LAMP reaction buffer. Inner images are the corresponding photos of PC and NC samples. (**C**) Effect of different metal ion indicators on the colorimetric LAMP assay. (**D**) Effect of different Mg^2+^ ion concentration on the colorimetric LAMP assay.

Real-time fluorescence detection and quantification of nucleic acid amplification have been widely used to detect a variety of pathogens for disease diagnostics and monitoring^9–11^. However, fluorescence detection still relies on relatively expensive fluorescence filters and fluorescence dye, which limits its potential for POC diagnostic applications. To simplify the optical detection and enable POC diagnostics, we adapted colorimetric LAMP strategy to quantify nucleic acids by monitoring color changes of LAMP reaction in real-time in the presence of a metal ion indicator (e.g., Eriochrome black T (EBT)). To achieve highly sensitive molecular detection, a synergistically enhanced colorimetric strategy^20^ has been applied in our real-time colorimetric LAMP assay by using non-buffered LAMP solution (Fig. 1A). Briefly, when blue EBT indicator was added to the LAMP reaction solution, it reacted with Mg^2+^ ions in LAMP reaction solution and formed red EBT-Mg^2+^ complex. During LAMP reaction, there were large amounts of pyrophosphate ion (PPi^4-^) and H^+^ ions generated as byproducts. On the one hand, the generated PPi^4-^ ions can react with the Mg^2+^ ions and form insoluble Mg_2_P_2_O_7_, which releases the blue EBT indicator and results in color change from red to blue. One the other hand, the decreasing pH value caused by produced H^+^ ions in the non-buffered LAMP solution can further accelerate the release of the blue EBT from the red EBT-Mg^2+^ complex. As shown in Fig. 1B, the synergistic effect of PPi^4-^ and H^+^ ions with the non-buffered LAMP solution resulted in a hue value difference (∆Hue) of 110° between negative control and positive control, which was approximately 83.33% improvement compared to that of commercially available NEB LAMP buffer solution (New England Biolabs) (∆Hue = 60°). By real-time monitoring the color change (hue value) of LAMP reaction, nucleic acid target can be detected and quantified without need for fluorescence dye and fluorescence detector (Fig. 1A).

There are many different indicators which have been used for colorimetric detection of metal ions (e.g., Mg^2+^, Ca^2+^)^25–27^. To optimize our real-time colorimetric LAMP assay, we first evaluated and tested different metal ion indicators and their mixture. As shown in Fig. 1C, the EBT indicator generated the most significant hue difference (∆Hue = 110°), three times higher than that of HNB indicator (∆Hue = 36°) used in the previous study^20^, which is crucial to developing a highly sensitive, reliable, quantitative nucleic acid detection by using a simple unmodified smartphone. In addition, the EBT indicator showed excellent stability at room temperature and no obvious hue value change was observed in LAMP reaction products after 5-week storage even if exposed under daylight (Figure S2). Since Mg^2+^ ion plays a critical role in enzymatic amplification as a cofactor/ catalyzer and generation of the EBT-Mg^2+^ complex, we further determined the effect of different Mg^2+^ ion concentrations (ranging from 4 to 10 mM) on the colorimetric LAMP detection. As shown in Fig. 1D, 8 mM Mg^2+^ ions showed the most significant hue value difference between positive and negative samples. Too high Mg^2+^ ions concentration can influence the stability of individual base pairs and induce the mismatch of base pairs, resulting in false positive signals due to non-specific amplification^28,29^. Therefore, 8 mM Mg^2+^ ions were used for the rest of the experiments.

**Figure 2:**
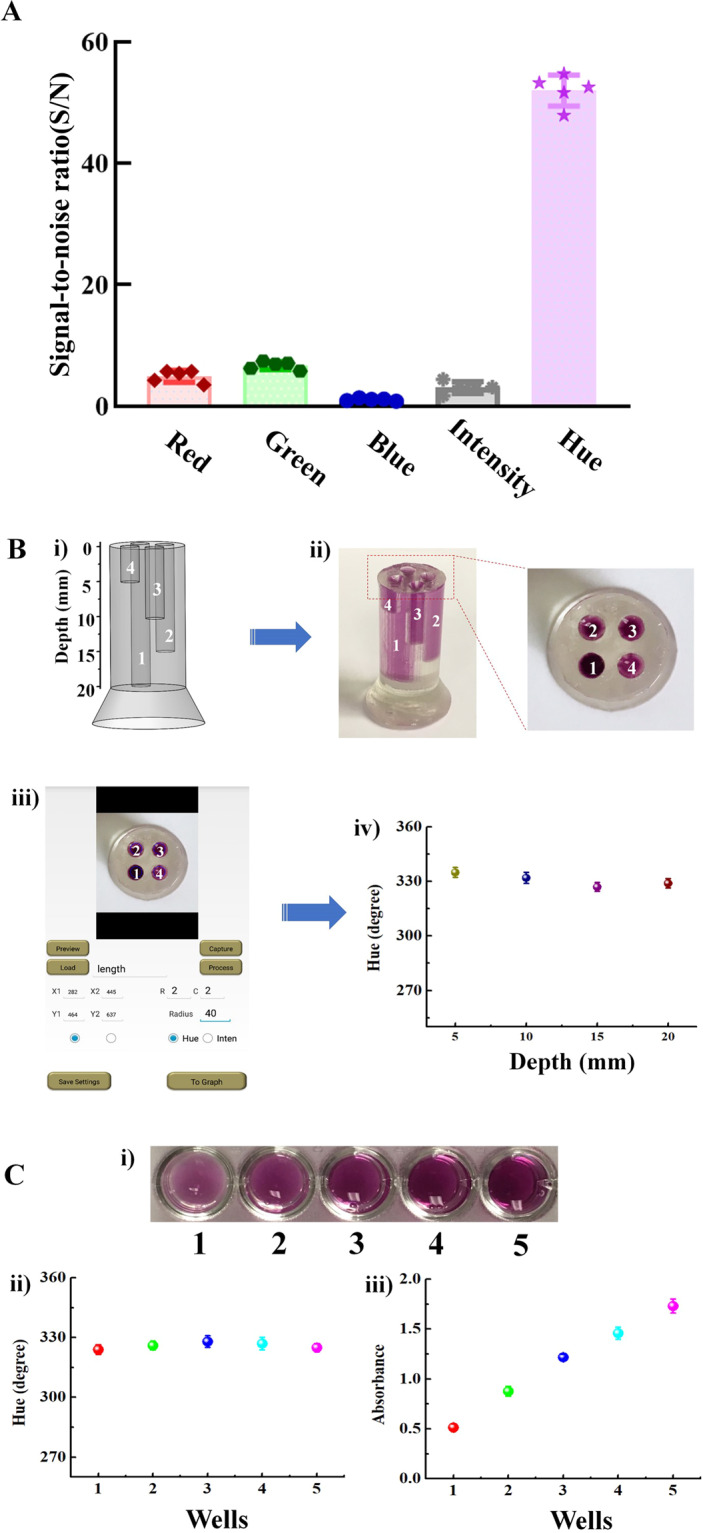
Hue-based colorimetric analysis. (**A**) Comparison of signal-to-noise (S/N) of RGB model and hue value analysis (HSI model) in colorimetric LAMP assay. (**B**) 3D printed device containing four cylindrical chambers with various depths (5, 10, 15 and 20 mm) for hue value measurement of the EBT indicator: i) schematic illustration of the 3D printed device; ii) a photograph of the device filled with the EBT indictor; iii) the interface of our smartphone app for hue value quantitative measurement and iv) Hue value (in degree), as a function of the depths of the chambers (in mm). (**C**) Comparison of hue value detection and absorbance measurement of the EBT indicator solution with various volumes in 96-well plate. (n = 5).

Different color models have been developed for color analysis of colorimetric assay^30–33^. Among them, RGB (red, green and blue) color model is one of the most commonly used approaches for the colorimetric analysis^30,31^. HSI (hue, saturation and intensity) color model is another widely used one for colorimetric assay and computer graphics. In the HSI color model, hue value can be measured as an angle in the color with a range of values between 0 and 360° (Figure S3), and calculated by RGB values^33^. Here, we compared the performance of these two-color models in our real-time colorimetric LAMP assay. As shown in Fig. 2A, the signal-to-noise ratio (S/N) of our hue value analysis (HSI model) was ~ 50, which was more than 10 times higher than that of RGB model and light intensity detection. To further determine the robustness of the HSI model in the colorimetric analysis, we designed and fabricated a 3D printed device containing four independent cylindrical chambers with various depths (5, 10, 15 and 20 mm) (Fig. 2B(i),(ii)). The EBT indicator was added into the chambers as a color indicator for hue value measurement by our smartphone app (Fig. 2B(iii)). As shown in Fig. 2B(iv), hue value measurement was totally independent on the depths of the chambers, which may be contributed that the hue value measurement is only dependent on dominant wavelength, not the intensity^34^. Unlike traditional absorbance or fluorescent detection, hue-based color analysis is not sensitive to sample volume change (Fig. 2C). This is important for microfluidic-based diagnostic detection because most of microfluidic chambers have small volumes and their microfluidic chambers may significantly vary due to fabrication errors. Further, previous study reported that hue value measure was insensitive to various ambient light condition^20^. Therefore, all these unique characteristics of hue-based colorimetric analysis make it ideal to develop simple, sensitive, reliable, real-time colorimetric LAMP assay for nucleic acid quantification.

**Figure 3:**
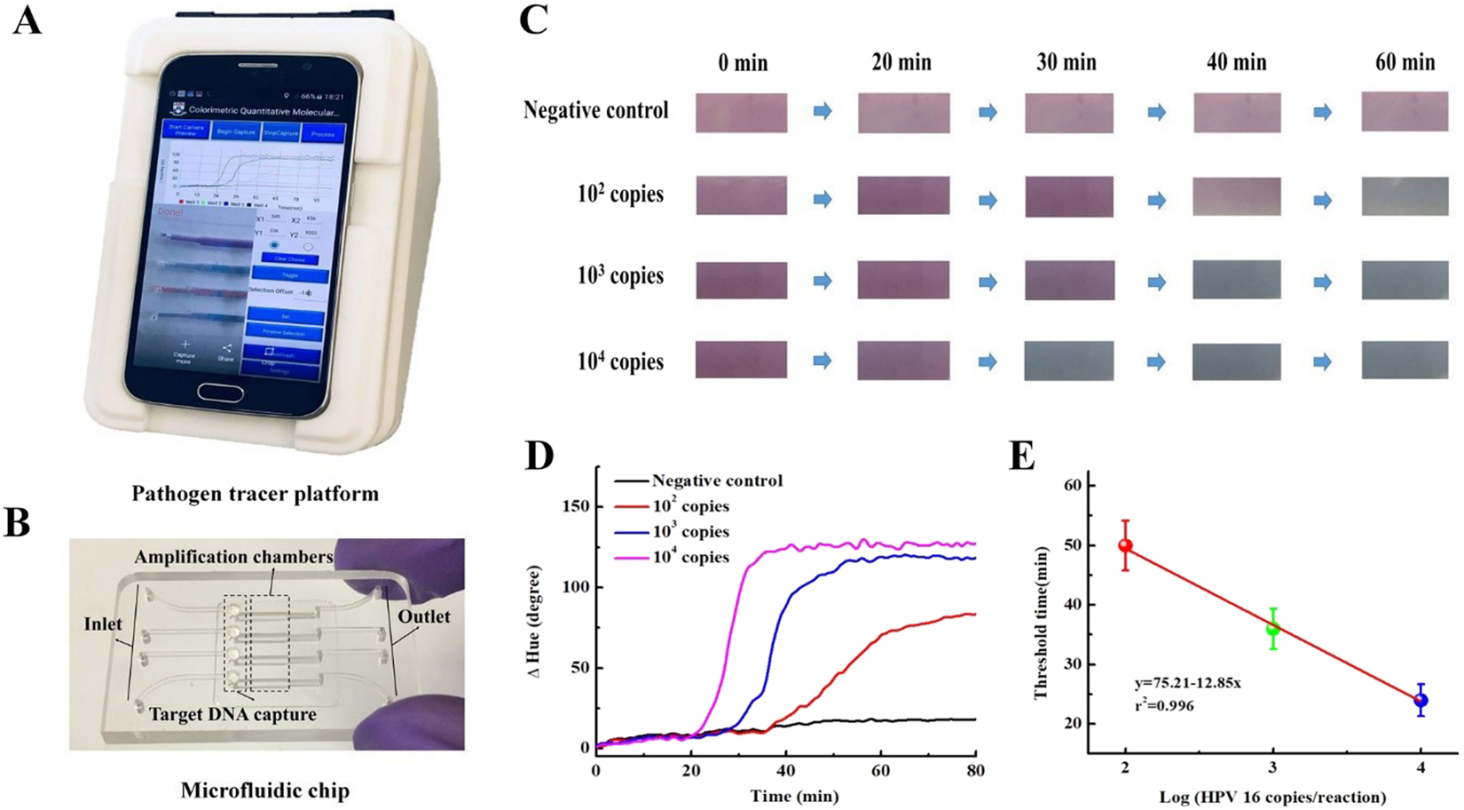
Hue-based quantitative molecular detection by our SCPT platform. (**A**) A photograph of our SCPT platform. (**B**) A photograph of our microfluidic chip. (**C**) A sequence of optical images obtained by smartphone camera during colorimetric LAMP assay of HPV 16 DNA ranging from 0 to 10^4^ copies per reaction. The images were taken at 0, 20, 30, 40 and 60 min after the start of LAMP incubation. (**D**) Real-time hue value change (∆Hue) monitoring of LAMP amplification. (**E**) Threshold time Tt (in minutes), as a function of the HPV 16 DNA concentration (expressed in terms of HPV 16 DNA copies per reaction).

The portable SCPT platform (Fig. 3A and Figure S1) was 3D printed with Acrylonitrile Butadiene Styrene (ABS) material and the microfluidic chip (Fig. 3B) was featured four independent reactors for simultaneously testing four samples. The porous silica membrane embedded into the individual reactors can capture and concentrate nucleic acid from raw samples, enabling elution-free, on-chip nucleic acid amplification and quantification. The app took images of the LAMP reactors at a constant interval (e.g., once a minute) and constructed real-time ∆Hue curves as functions of incubation time (Figure S4). At the end of the test, to quantify nucleic acid, the threshold times were calculated and a calibration formula with linear relationship between the threshold time and the log of the nucleic acid could be obtained by simultaneously testing two known concentration samples (Figure S4B,C). The calibration formula could also be stored by the app for future nucleic acid quantification if needed. As shown in Figure S4D, for remote health monitoring and disease mapping, GPS coordinates of the test location were recorded and saved in the server along other detection information (e.g., quantitative results, test time).

Figure 3C and Video S2 showed real-time hue monitoring of four independent LAMP reactors containing 0 (negative control), 10^2^, 10^3^, and 10^4^ copies HPV 16 DNA. To obtain a zero-hue value baseline, we first imaged the background at the start of the monitoring process and calculated its hue value. Then, we plotted the hue value change (ΔHue) by using the background hue value to subtract that of the subsequent images. As shown in Fig. 3D, the higher the target DNA concentration is, the earlier the hue value change (ΔHue) curve increases above the baseline. In the negative control reactor, the hue value change (ΔHue) remains nearly constant at the baseline during the entire LAMP incubation. The threshold time (*T*_*t*_) was defined as the reaction time that elapses until the hue value change increases by ∼20% above the baseline level. Figure 3E depicts the threshold time *T*_*t*_ (min) as a function of the target concentration C (HPV 16 DNA per reaction). Our experiments indicated that real-time hue-based LAMP assay on our SCPT platform could quantitatively detect less than 100 copies HPV 16 DNA, which is comparable to that of real-time fluorescence LAMP assay (Figure S5) using the state-of-art PCR machine.

**Figure 4:**
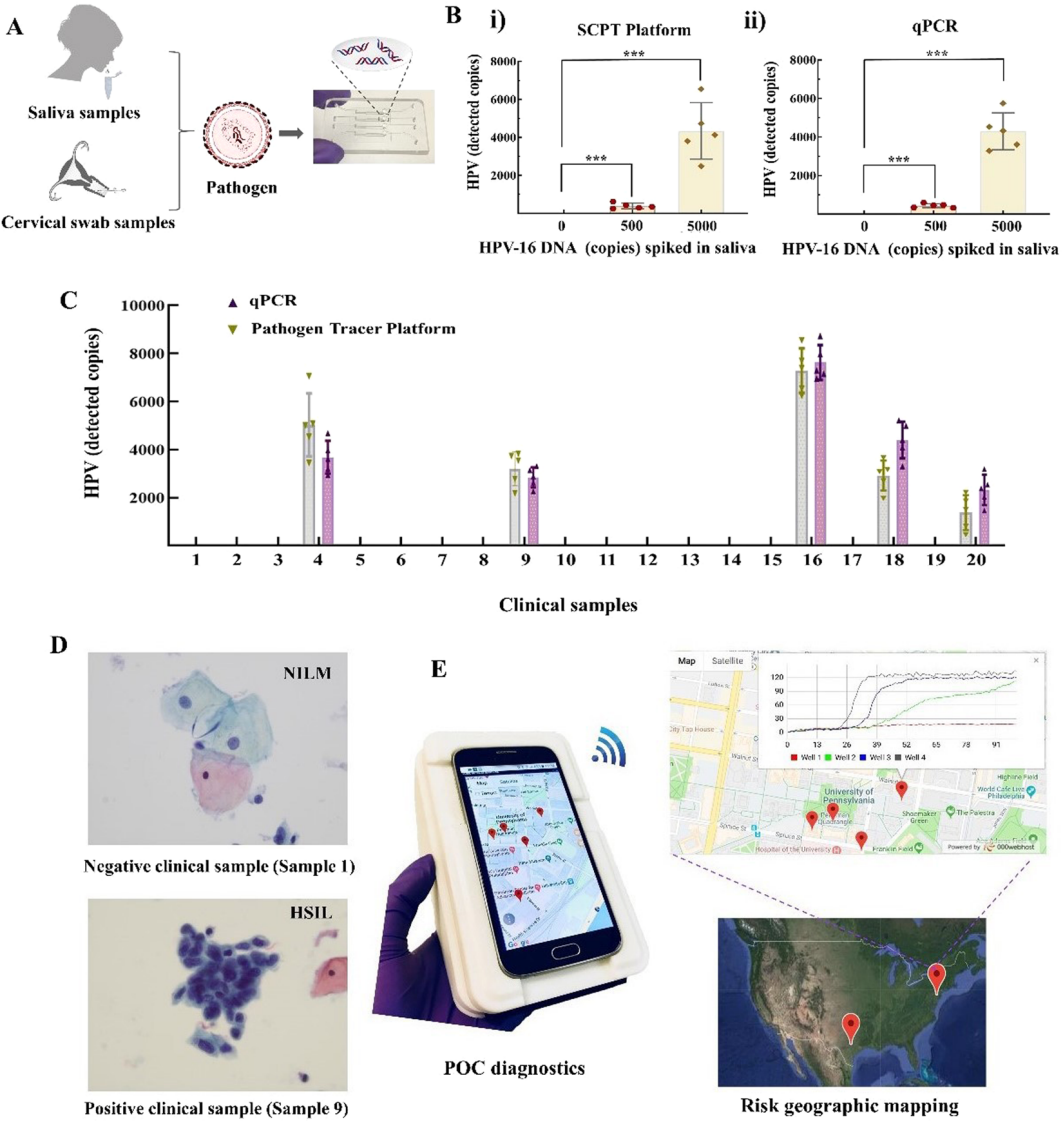
Clinical validation and smart, connected health monitoring on the SCPT platform. (**A**) A process of on-chip nucleic acid extraction from different clinical samples. (**B**) Comparison of HPV 16 DNA quantitative detection in spiked saliva samples with: i) the SCPT platform and ii) qPCR method. (**C**) Quantitative detection of clinical vaginal swab samples by the SCPT platform and qPCR method. (**D**) Representative images of Pap smear results for negative clinical sample (clinical sample 1 in Fig. 4C) and positive clinical sample (clinical sample 9 in Fig. 4C). NILM and HSIL stand for High-Grade Squamous Intraepithelial Lesion and Negative for Intraepithelial Lesion or Malignancy, respectively. (**E**) Smart, connected disease monitoring and pathogen tracking by the SCPT platform.

Clinical samples detection was performed on our custom-made microfluidic chip (Fig. 4A). Nucleic acids in lysed sample can be extracted and purified when it flows through the silica isolation membranes of the microfluidic chip^22^, decoupling sample volume from the reactor volume and enabling highly sensitive molecular diagnostics. To evaluate the performance of our diagnostic platform, we detected various pathogens (e.g., HPV, HIV) in different human samples (e.g., saliva, vaginal swab and plasma). First, we quantified the known HPV 16 DNA spiked in the saliva samples by using our SCPT platform. As shown in Fig. 4B, the quantitative detection capability of our SCPT platform was comparable to that of the state-of-art qPCR method. Next, we investigated its clinical application by testing 20 clinical vaginal swab samples. The clinical swab samples were collected at the Hospital of the University of Pennsylvania under the approved IRB protocol. As shown in Fig. 4C, the detection results of clinical swab samples with our SCPT platform were consistent with those of the qPCR method. All 15 negative samples confirmed by the qPCR method showed negative signal with the SCPT platform, and 5 positive samples were quantitatively detected by our SCPT platform with an excellent agreement to the qPCR method. Compared to conventional qPCR method, the SCPT platform has a much shorter turnaround time (~1 hour) and eliminates the need for expensive equipment (e.g., real-time PCR machine). Figure 4D showed representative images of Pap smear results of negative clinical sample (clinical sample 1 in Fig. 4C) and positive clinical sample (clinical sample 9 in Fig. 4C). To further demonstrate versatility of our SCPT system, we quantitatively detected HIV RNA in plasma samples and achieved a sensitivity of 100 copies HIV RNA per test (Figure S6). Therefore, all these results showed that our SCPT platform was suitable for simple, rapid, POC molecular detection without need for expensive equipment and professional personnel.

The surveillance of the emerging infectious diseases (EIDs) spread is vital for early identification of pathogens and assessment of preventive care^35^. To demonstrate the connected health monitoring capability of our SCPT platform, a custom website was created to provide real-time reporting, quantitative detection, evidence preservation, and spatial analysis from the SCPT platform (Figs. 4E and S4D). When desired, the testing results along with patients’ information (e.g., age, gender, and occupation) can be wirelessly transmitted to a remote server and made available to the patient’s doctor and public health officials, enabling remote clinical diagnostics and real-time epidemic surveillance. The coordinates of infectious disease cases can be recorded and stored in the custom website, allowing to accurately and timely monitor the risk and progress of disease. Furthermore, global disease mapping coupled with spatial big data analysis will enable global disease forecasting and epidemic risk assessment. Therefore, our SCPT diagnostic platform, in combination with web-based surveillance, provides a new paradigm for global disease surveillance and mobile health monitoring.

## Discussion

Cancer and infectious diseases have become the leading causes of death and posed a considerable threat to global health. Rapid detection of these diseases and accurate identification of their causative pathogens are crucial in disease prevention, treatment, and monitoring. We have developed a mobile molecular diagnostic platform for quantification of HPV-16 DNA in saliva and clinical cervical swab samples, and HIV RNA in plasma with a sensitivity of less than 100 copies per test, comparable to that of the real-time qPCR method. The mobile SCPT platform is simple, compact and user-friendly, and can be operated by minimally-professional personnel.

Several unique features make our SCPT platform suitable for point of care diagnostics and remote health monitoring in resource-limited areas. First, the real-time hue-based LAMP assay is inherently robust, which simplifies the optical detection and enables us to quantify nucleic acid biomarkers directly by an unmodified smartphone. Especially, a synergistically enhanced strategy has been adapted for highly sensitive colorimetric LAMP assay. Second, our SCPT platform is simple, compact, and cost-effective. The platform includes simple electronics and can be powered by lithium battery. The plastic chip for nucleic acid extraction and isothermal amplification is made of low-cost PMMA material and its cost can be further reduced if mass production (e.g., injection molding) is used. Especially, the smartphone is used to take photo, processing images, analyzing data and reporting results, eliminating the need for expensive equipment. Third, the SCPT platform is connected and ready for remote health monitoring and epidemic surveillance. By taking advantage of wireless internet, the test result along with other information (e.g., locations, test time) can be uploaded to the custom website by smartphone, and, if needed, shared with the patient’s doctor and public health officials, providing a new paradigm for mobile health monitoring and epidemic analysis.

HPV and HIV were utilized as model pathogens to demonstrate the feasibility of our SCPT for its application in connected health monitoring and epidemiological surveillance. However, the SCPT platform is generic and can be extended to detect other pathogens by slightly modifying the primers. More LAMP reactors can also be integrated in a single chip for multiplex detection of different pathogens because of the wide field of view of smartphone camera. Although the current study is not designed to focus on the surveillance of the emerging infectious diseases, the mobile connectivity capability of our diagnostic platform, coupled with web-based surveillance, makes it suitable for future disease spatial mapping and epidemic analysis. Thus, such rapid and cost-affordable mobile molecular diagnostic technology is envisioned for a wide variety of applications ranging from early disease detection, remote healthcare monitoring, to global epidemic surveillance.

## Supplementary information

Supplementary Information.

Supplementary Video S1.

Supplementary Video S2.
