# Supplementary material for: Real-time Colorimetric Quantitative Molecular Detection of Infectious Diseases on Smartphone-based Diagnostic Platform
Source: Sci Rep. 2020 Jun 2;10:9009. doi: 10.1038/s41598-020-65899-w (PMC7265458; doi:10.1038/s41598-020-65899-w)
Supplement: Supplementary file 1 — Supplementary Information. [file 41598_2020_65899_MOESM1_ESM.docx]

**
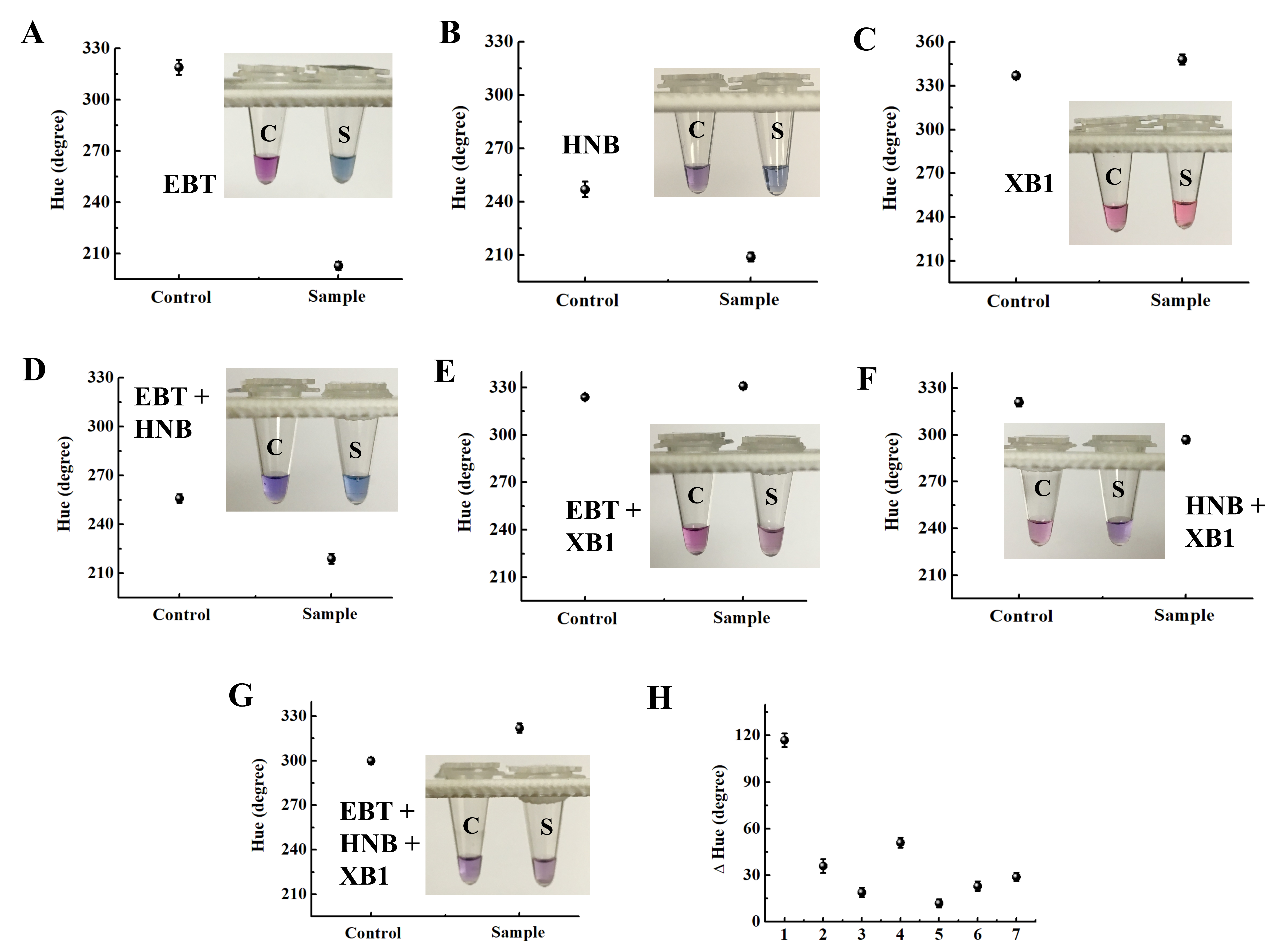
Supporting Information**

**Real-time Colorimetric Quantitative Molecular Detection of Infectious Diseases on Smartphone-based Diagnostic Platform**

Kun Yin, ^a,b^ Vikram Pandian, ^b^ Karteek Kadimisetty ^b^ Xin Zhang,^b^ Carlos Ruiz,^b^ Kumarasen Cooper, ^c^ and Changchun Liu *^*^*^,a,b^

^a^ Department of Biomedical Engineering, University of Connecticut Health Center, 263 Farmington Avenue, Farmington, CT 06030, USA

^b^  Department of Mechanical Engineering and Applied Mechanics, University of Pennsylvania, 220 South 33rd St. Philadelphia, Pennsylvania 19104-6315, USA

^c^ Department of Pathology and Laboratory Medicine, University of Pennsylvania, 3400 Spruce St. Philadelphia, PA 19104, USA

Corresponding Author

Changchun Liu:

*E-mail: chaliu@uchc.edu

Table S1 Primers for HPV 16 and HIV isothermal amplification

| **Target** | **Primer** | **Primer Sequence (5'-3')** |
| --- | --- | --- |
| HPV 16 ^[^[^1^](#_ENREF_1)^]^ | F3  B3  FIP  BIP  Loop F | CAAATTATTTTCCTACACCTAGTGG  GTCATAACGTCTGCAGTTAAGG  GTGGCCCTGTGCTCGTTGTCTATGGTTACCTCTGATGCC  CACGCAGTACAAATATGTCACCCCATGTCGTAGGTACTCC  GCTGCCATATCTACTTCAGAAACTACA |
| HIV ^[^[^2^](#_ENREF_2)^]^ | F3  B3  FIP  BIP  Loop F  Loop B | ATTATCAGAAGGAGCCACC  CATCCTATTTGTTCCTGAAGG  CAGCTTCCTCATTGATGGTTTCTTTTTAACACCATGCTAAACACAGT  TGTTGCACCAGGCCAGATAATTTTGTACTGGTAGTTCCTGCTATG  TTTAACATTTGCATGGCTGCTTGAT  GAGATCCAAGGGGAAGTGA |


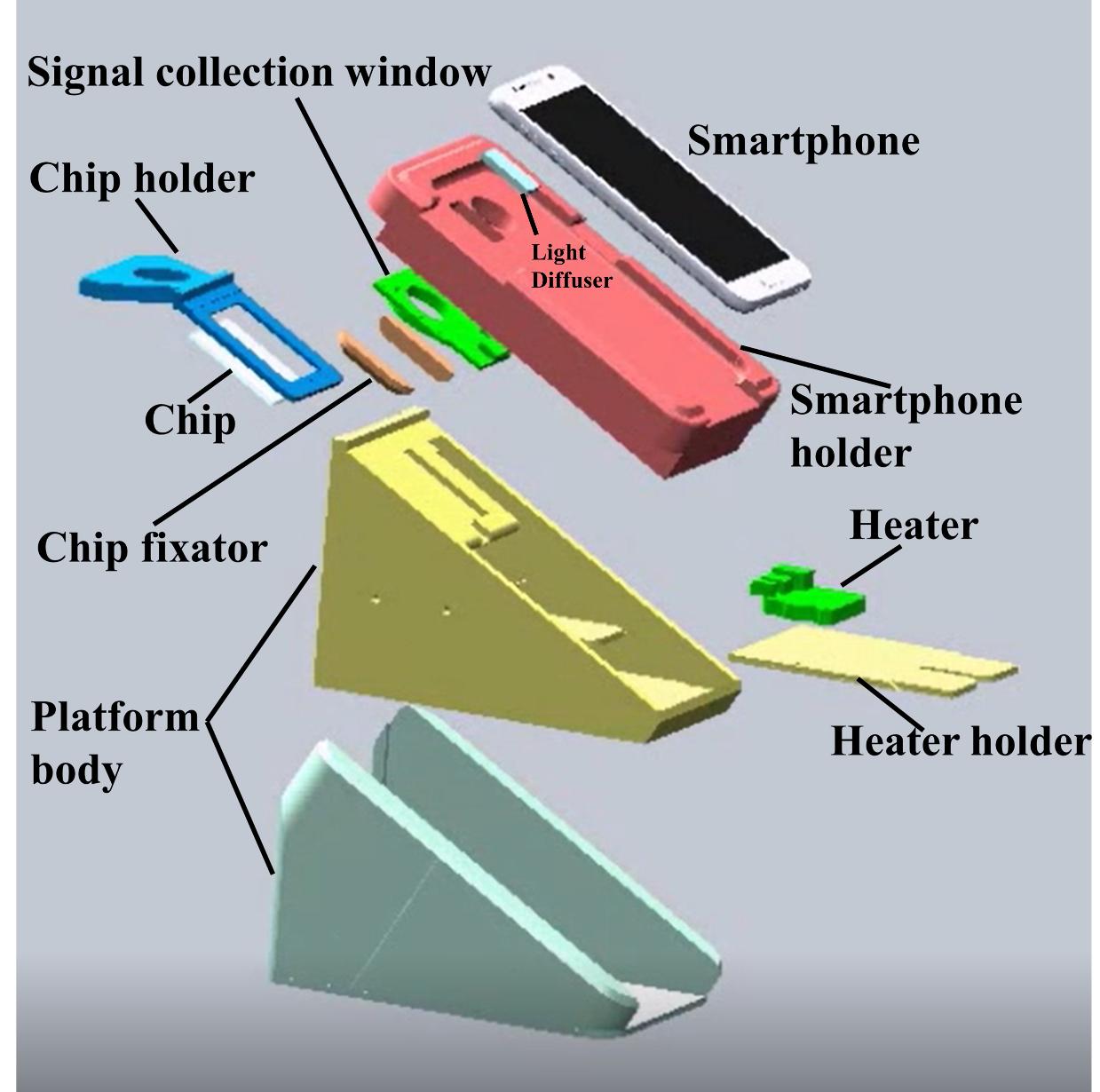


**Figure S1.** Exploded view of our smart connected pathogen tracer (SCPT) platform.


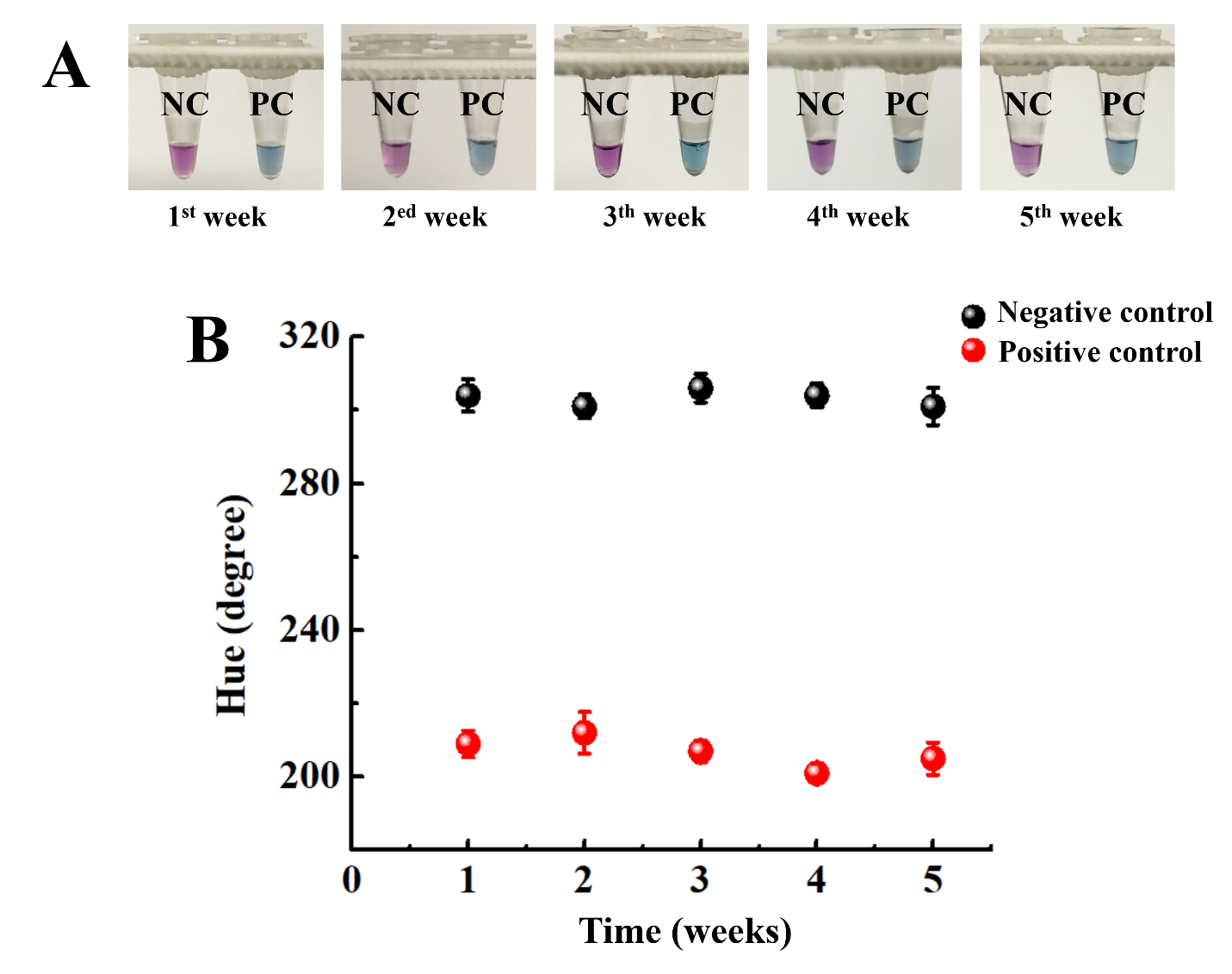


**Figure S2.** Stability evaluation of Eriochrome black T (EBT) indicator after colorimetric LAMP assay in non-buffered LAMP solution. A) Colorimetric LAMP assay of PC (10^3^ copies of HPV 16 DNA) and NC (no template DNA) samples in non-buffered LAMP solution stored at room temperature for different times (0 to 5 weeks). B) Hue value analysis of the corresponding samples in **Figure S2A**. Error bars denote s.d. (n=3).


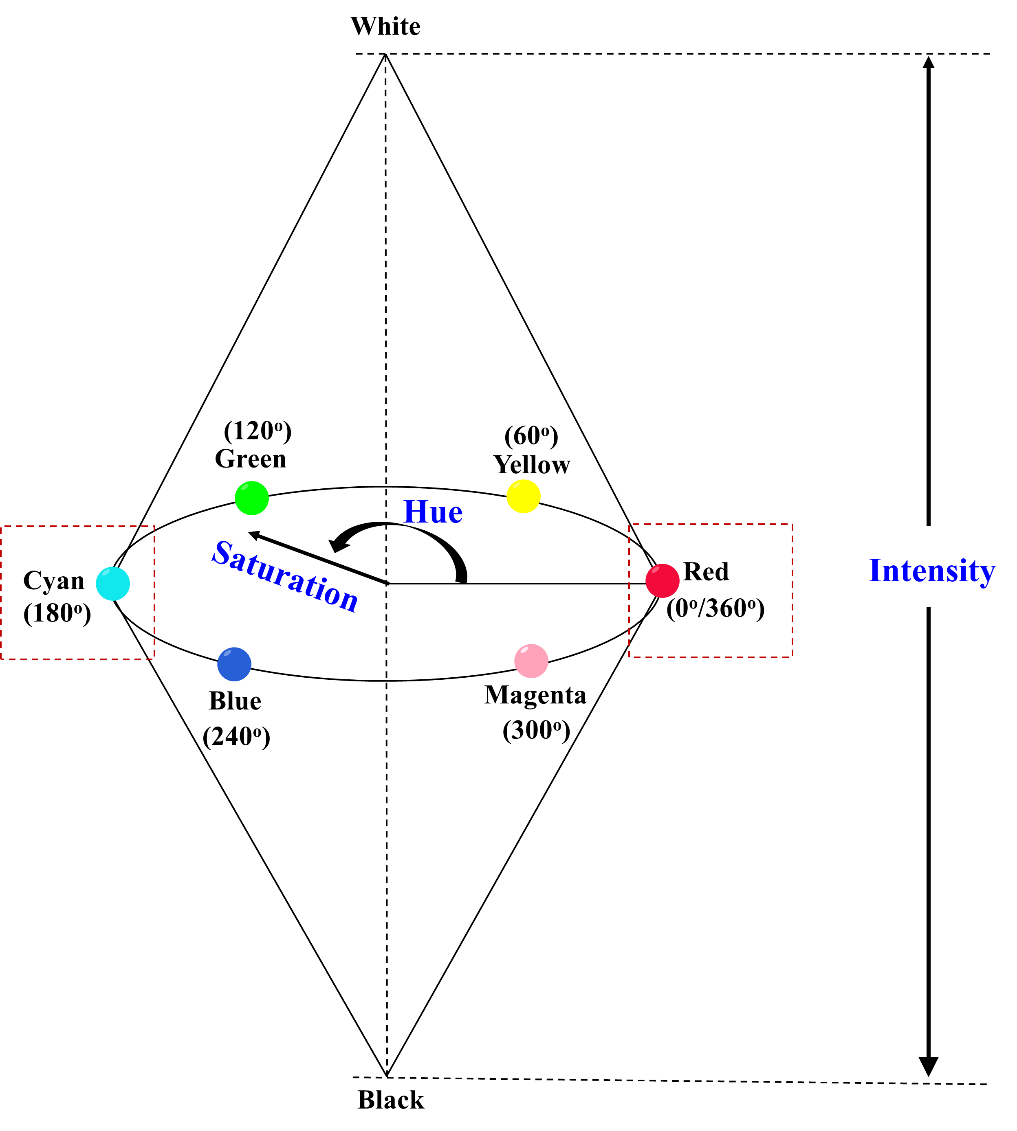


**Figure S3.** HSI color space model. The HSI color model uses hue (H), saturation (S) and intensity (I) to represent color images. Hue value is measured in degrees with a range of values between 0 and 360°.

**
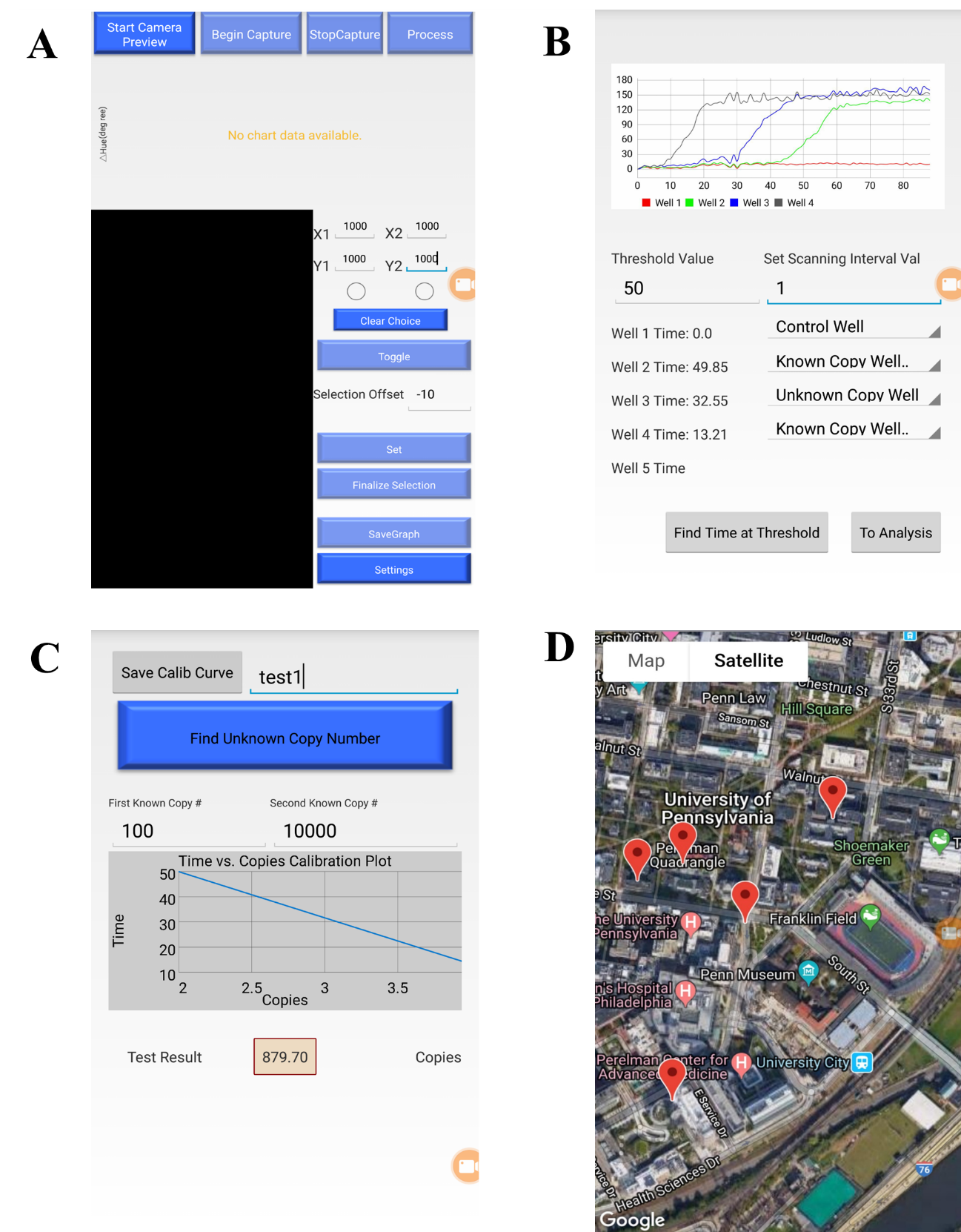
**

**Figure S4.** Screenshots of the smartphone app interface. A) Signal read-out and real-time monitoring, B) Real-time hue value change (ΔHue) LAMP curves, C) Calibration curve for nucleic acid quantification, and D) Web-based mapping of test results with the SCPT platform.


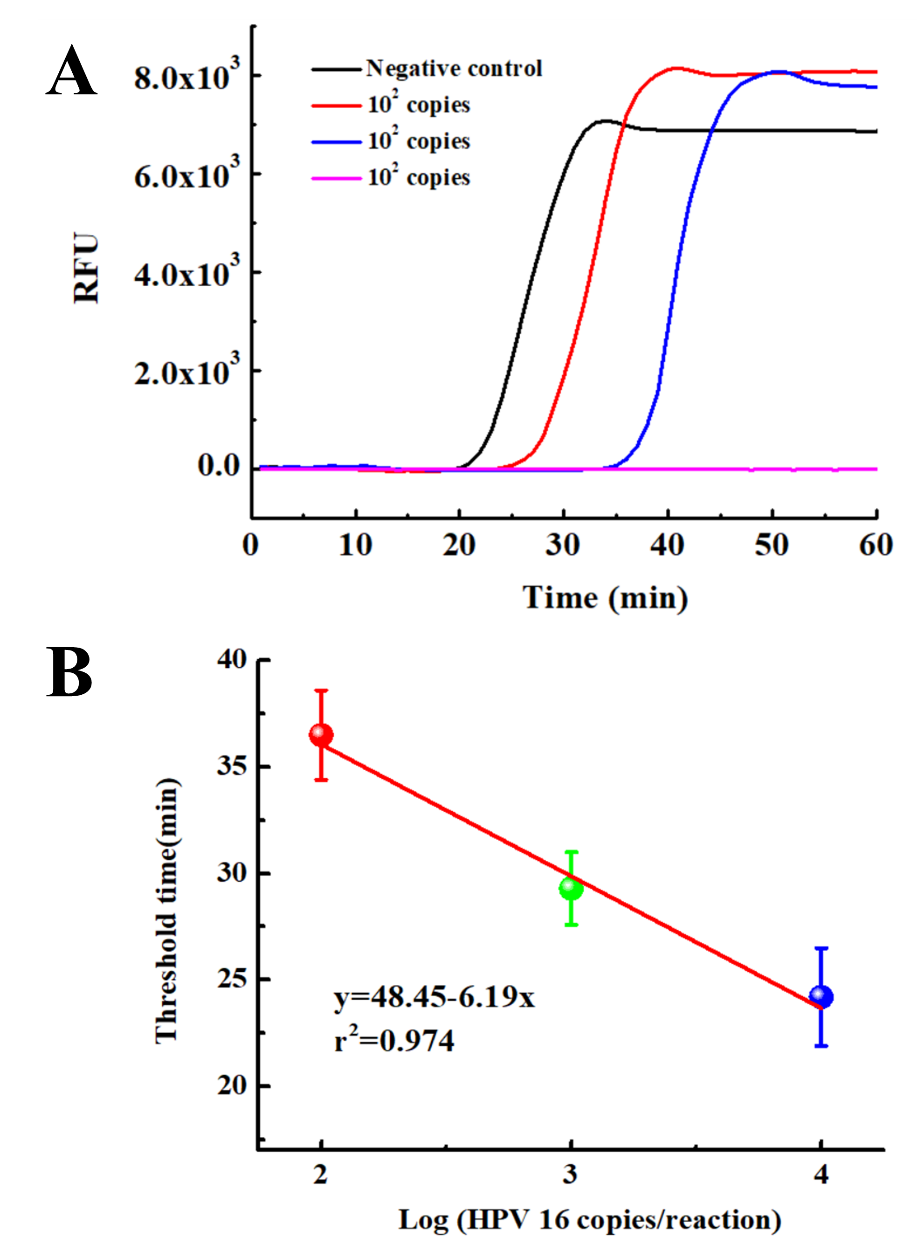


**Figure S5**. A) Real-time fluorescence monitoring of LAMP amplification of 0 (negative control), 10^2^, 10^3^, and 10^4^ copies of HPV 16 DNA per reaction on benchtop PCR machine. B) The threshold time (min) as a function of the HPV 16 DNA concentration (copies per reaction). Error bars denote s.d. (n=3).


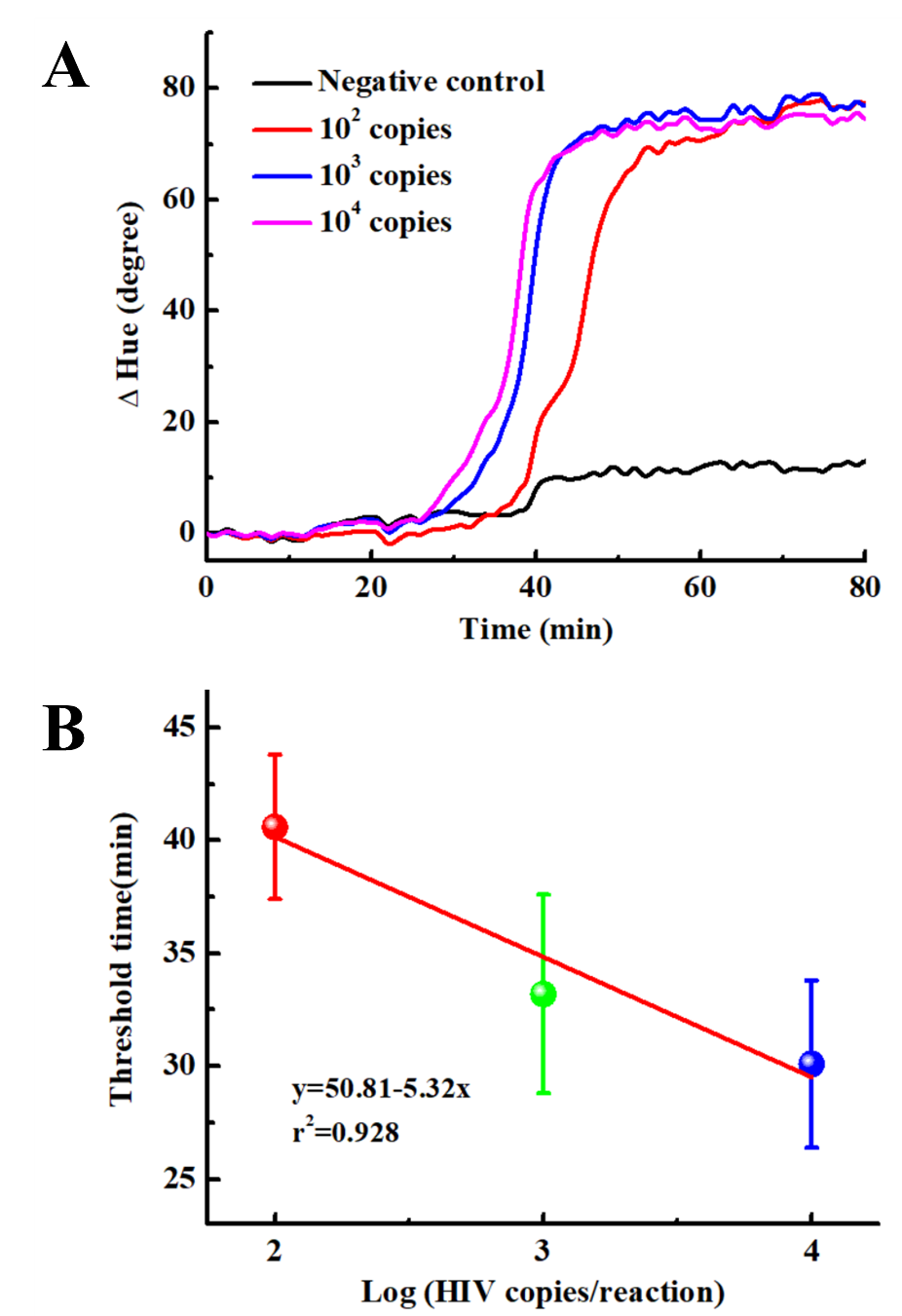


**Figure S6.** A) Real-time hue-based RT-LAMP amplification of 0 (negative control), 10^2^, 10^3^, and 10^4^ copies of HIV RNA per test on the SCPT platform. B) The threshold time (min) as a function of HIV RNA concentration (copies per reaction). Error bars denote s.d. (n=3).

[1] T. Satoh, K. Matsumoto, T. Fujii, O. Sato, N. Gemma, M. Onuki, H. Saito, D. Aoki, Y. Hirai, H. Yoshikawa, *Journal of virological methods* **2013**, *188*, 83-93.

[2] B. Sun, F. Shen, S. E. McCalla, J. E. Kreutz, M. A. Karymov, R. F. Ismagilov, *Analytical chemistry* **2013**, *85*, 1540-1546.
